# Infrapatellar plica injury: Magnetic resonance imaging review of a neglected cause of anterior knee pain

**DOI:** 10.4102/sajr.v25i1.1973

**Published:** 2021-02-19

**Authors:** Dharmendra K. Singh, Heena Rajani, Mukul Sinha, Amit Katyan, Saurabh Suman, Aayushi Mishra, Bibhu K. Nayak

**Affiliations:** 1 Department of Radiology, Vardhman Mahavir Medical College, Safdarjung Hospital, New Delhi, India; 2 Department of Sport’s Medicine, Vardhman Mahavir Medical College, Safdarjung Hospital, New Delhi, India

**Keywords:** MR imaging, knee, synovial plica, infrapatellar plica injury, anterior knee pain

## Abstract

Synovial plicae are normal remnants of synovial membranes within the knee joint cavity and are usually asymptomatic. Pathological infrapatellar plica, which is mostly due to plica injury, may be a potential cause of anterior knee pain, but is often overlooked and under-reported on magnetic resonance imaging (MRI). This pictorial review illustrates the MRI findings of infrapatellar plica injury and associated knee injuries, with emphasis on its differentiation from the mimics of plica injury.

## Introduction

Synovial plicae are normal folds of synovial tissue within a joint cavity, which represent normal remnants of synovial membranes during embryological development of the knee joint. There are four main plicae in the knee joint, namely infrapatellar plica (IPP) or ligamentum mucosum, suprapatellar plica, medio-patellar plica and lateral patellar plica, in decreasing order of occurrence.^1^ These may become symptomatic because of plica syndrome or injury. Although IPP is the most commonly encountered plica, present in about 65% of patients, traditionally, it is considered least likely to be symptomatic.^2,3^

This review illustrates a series of cases where patients presented to the Radiology department of a tertiary care hospital with knee pain, often with a history of known trauma. The cases demonstrated abnormality along the course of the IPP, interpreted as either plica *sprain* or *tear*.

Magnetic resonance imaging (MRI) was performed on a 3T unit (Discovery MR 750W 3T- GE Healthcare) in all cases. The T1W images were acquired in the coronal plane; PDFS (proton density fat-saturated) images in the axial, oblique sagittal and coronal planes; and T2W images were acquired in the oblique sagittal plane.

## Anatomy and magnetic resonance imaging appearance of the normal infrapatellar plica

**FIGURE 1:**
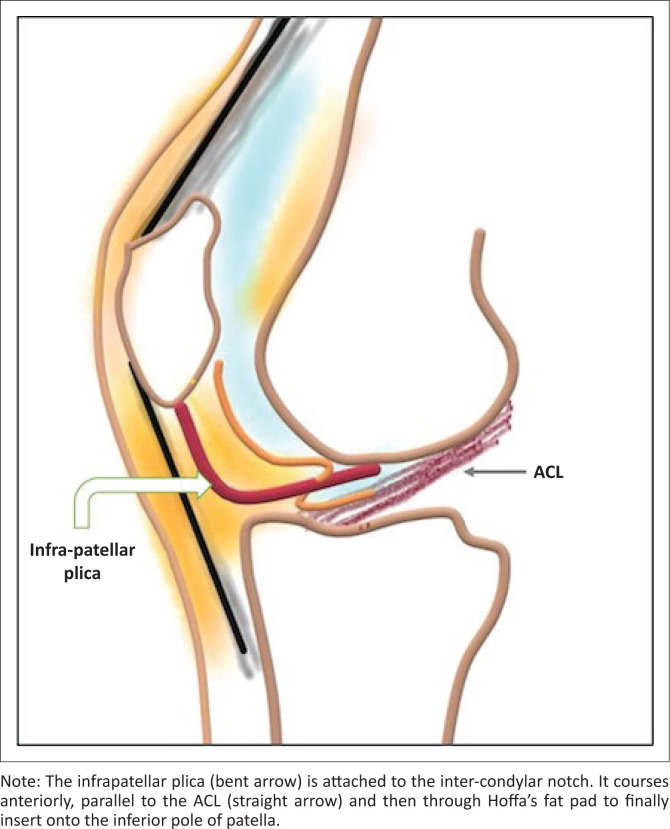
Schematic diagram of the anatomy of the infrapatellar plica and anterior cruciate ligament (ACL).

The IPP originates from the anterior part of inter-condylar notch and courses parallel to the anterior cruciate ligament (ACL) along its anterior aspect. It further courses through Hoffa’s fat pad to finally insert on the inferior pole of patella (Figure 1). Its dimensions vary from being very thin to being as thick as the ACL. However, its course in Hoffa’s fat pad is more delicate. It may attach to the transverse inter-meniscal ligament before coursing through Hoffa’s fat pad.^4^ It is classified anatomically based on the degree of regression shape as: (1) having a vertical septum, (2) separate from the ACL, (3) split or bipartite or (4) fenestrated.^1^

The antero-medial meniscofemoral ligament (AMMFL) is an accessory ligament with a prevalence of 0.77% in knee MRI studies. Although its course in the inter-condylar notch may be similar to the IPP, it attaches to the anterior horn of the medial meniscus and does not course through Hoffa’s fat pad. Injuries to the AMMFL are not clinically significant.^5^

**FIGURE 2:**
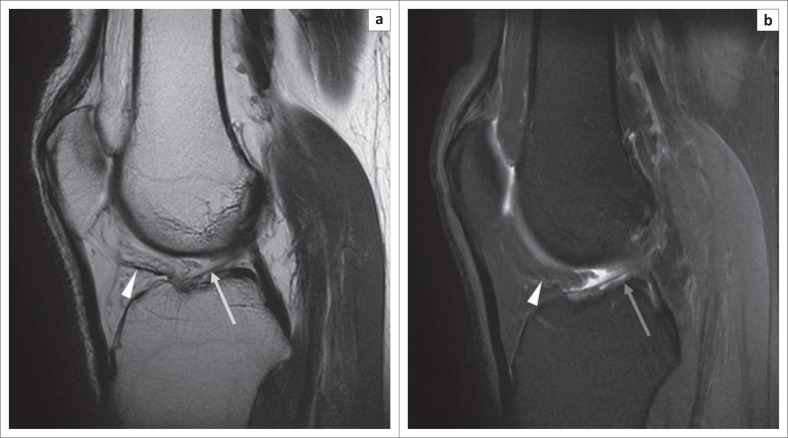
Magnetic resonance imaging of a normal infrapatellar plica: sagittal T2-weighted image (T2WI) (a) and sagittal proton density fat-saturated (PDFS) image (b) revealing the normal infrapatellar plica as a thin low-signal intensity structure anterior to anterior cruciate ligament in the inter-condylar notch (arrow) and in Hoffa’s fat pad (arrow head).

**FIGURE 3:**
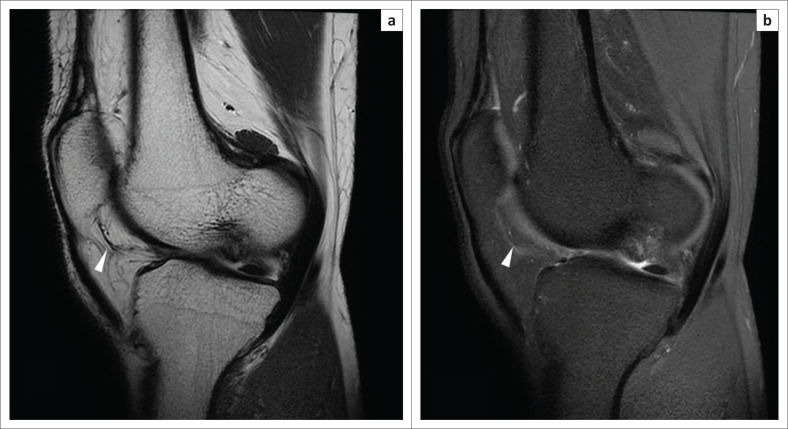
Magnetic resonance imaging of a normal infrapatellar plica: oblique sagittal T2WI (a) and oblique sagittal PDFS image (b) demonstrating the normal infrapatellar plica (arrow head) as a thin low-signal intensity structure within Hoffa’s fat pad, inserting into the inferior pole of patella.

On the MRI, the plicae appear as low-signal intensity linear structures on all pulse sequences (Figures 2 and 3).^4^ The surrounding Hoffa’s fat pad shows normal signal intensity.

## Causes of anterior knee pain

The spatial approach described by Flores et al.^6^ provides a systematic approach to anterior knee pain and is summarised in Table 1 under the four anatomic layers of the knee joint. It is beyond the scope of the present article to describe in detail the imaging findings of all the above-mentioned pathologies. In this review, we intend to emphasise on the imaging appearances of IPP injury.

**TABLE 1:** Spatial approach to anterior knee pain.^6^

Layer	Anatomic structure	Pathologies
1. Superficial soft tissue	Skin	Trauma, Inflammation
	Subcutaneous fat	Trauma, Inflammation
	Fascia	Trauma, Inflammation
	Prepatellar bursa and Superficial infrapatellar bursa	Bursitis
2. Extensor mechanism	Quadriceps tendon	Tear, Tendinosis
	Patella	Fracture, Mal-tracking, Tractional apophysitis (Sinding-Larsen-Johansson syndrome), Tumours, Osteomyelitis
	Patellar tendon	Tear, Tendinosis
	Tibial tuberosity	Fracture, Chronic avulsion injury (Osgood- Schlatter Disease)
	Medial and Lateral patellar retinaculum	Injury
3. Intracapsular (extra-synovial)	Infrapatellar fat pad	Infrapatellar fat pad impingement syndrome (Hoffa’s Disease), Traumatic tears/ contusions, Post-surgical fibrosis, Para-articular chondroma, Ganglion cysts, Focal nodular synovitis
	Deep infrapatellar bursa	Bursitis
	Suprapatellar fat pad and Pre-femoral fat pad	Oedema, Fibrosis, Focal nodular synovitis
4. Intra-articular	Plicae	Plica syndromes, Inferior patellar plica tear
	Synovium	Arthritis, Focal nodular synovitis, Lipoma arborescens, Pigmented Villo-Nodular Synovitis (PVNS), Synovial chondromatosis
	Articular cartilage	Patello-femoral osteoarthrosis

*Source*: Flores D, Mejía Gómez C, Pathria M. Layered approach to the anterior knee: Normal anatomy and disorders associated with anterior knee pain. RadioGraphics. 2018;38(7):2069–2101. https://doi.org/10.1148/rg.2018180048

## Plica injury

Plica tear is known to cause anterior knee pain, if it is complicated by haemorrhagic rupture, thickening or fibrosis.^2,3,7^

### Biomechanics of infrapatellar plica injury

The IPP has two components: an anterior component within Hoffa’s fat pad and a posterior component located anterior to the ACL. At arthroscopy, only the portion of the IPP anterior to the ACL is visualised and normally remains horizontal in both flexion and extension. On the contrary, radiologically appreciable IPP sprain or tear is detected in the portion of the IPP within Hoffa’s fat pad. It is noteworthy that a radiologically abnormal plica in Hoffa’s fat pad, detected by MRI, may not be symptomatic until it alters the position of the IPP anterior to ACL on dynamic arthroscopic evaluation, appearing horizontal on flexion but vertical on extension. This alteration in dynamics, presumably because of fibrosis or stiffness in the IPP, ultimately causes chondral lesions on the superior portion of the inter-condylar notch and patellofemoral sulcus as a result of friction and compression forces, consequently resulting in chronic anterior knee pain.^8^

### Magnetic resonance imaging appearance of infrapatellar plica injury

An injured or diseased IPP is suggested when a significant amount of curvilinear high T2-signal is seen along the expected course of the IPP in Hoffa’s fat pad, or if a markedly thickened plica is visualised, or both.^3,9^ Sagittal PDFS is the minimum sequence required for the optimal evaluation of an IPP injury.

**FIGURE 4:**
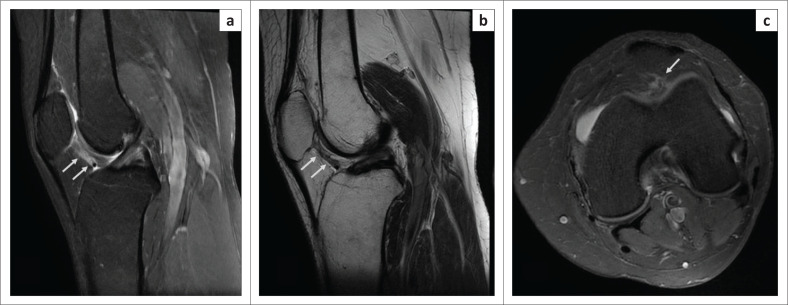
Magnetic resonance imaging (MRI) of infrapatellar plica sprain in a 50-year-old female with a history of twisting the knee: oblique sagittal PDFS image (a), T2WI (b) and axial PDFS image (c) indicate hyperintense signal along the entire course of infrapatellar plica (arrow), from the transverse inter-meniscal ligament to the inferior pole of patella, with its margins appearing ragged. Note the signal is less hyperintense than that of the synovial fluid. No other abnormality was noted on the patient’s knee MRI.

**FIGURE 5:**
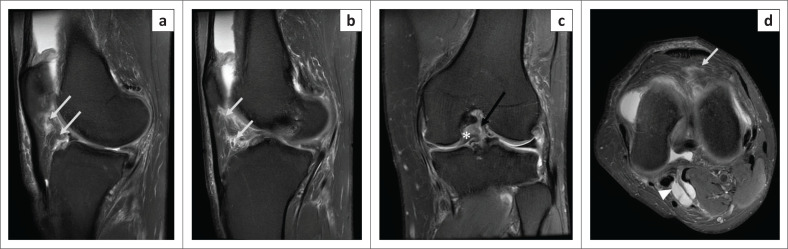
Magnetic resonance imaging of infrapatellar plica sprain in a 45-year-old male with a history of a fall: serial PDFS oblique sagittal images (a and b), oblique coronal PDFS (c) and axial PDFS (d) demonstrates hyperintense signal along the entire course of infrapatellar plica (white arrows in a, b and d) having ragged margins, with oedema in the surrounding Hoffa’s fat pad and oedema in the prepatellar subcutaneous plane (d). Note mild sprain in the middle third of the postero-lateral bundle of the anterior cruciate ligament in (c) (black arrow), associated bucket handle tear of the medial meniscus with the displaced fragment lying within the inter-condylar notch (c) (asterisk), small Baker’s cyst posteriorly (d) (arrow head).

**FIGURE 6:**
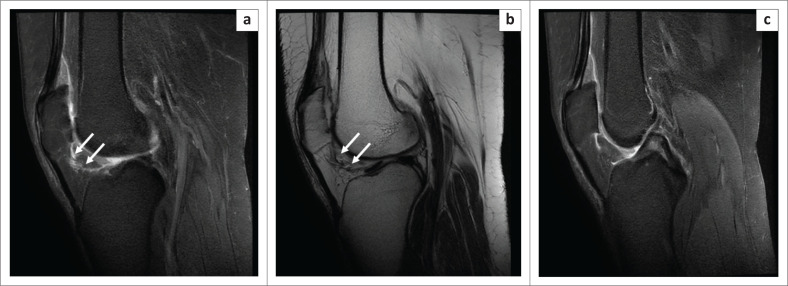
Magnetic resonance imaging of an infrapatellar plica tear in a 53-year-old female with a history of anterior knee pain: oblique sagittal PDFS image (a) and T2WI (b) reveal a hyperintense fluid collection along the entire course of the infrapatellar plica (arrows), with ragged margins. Oblique sagittal PDFS image (c) indicates a normal anterior cruciate ligament and posterior cruciate ligament in the patient.

**FIGURE 7:**
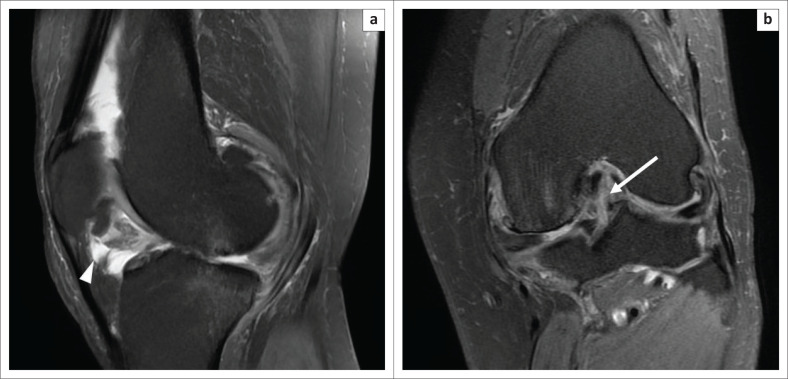
Magnetic resonance imaging of an infrapatellar plica tear in a 60-year-old female with a history of anterior knee pain: PDFS oblique sagittal image (a) shows a hyperintense fluid collection along the entire course of infrapatellar plica (arrow head) with oedema in the surrounding Hoffa’s fat pad. Oblique coronal PDFS (b) shows that the patient also had a high-grade partial thickness tear of the middle and upper thirds of both bundles of the anterior cruciate ligament (arrow).

**FIGURE 8:**
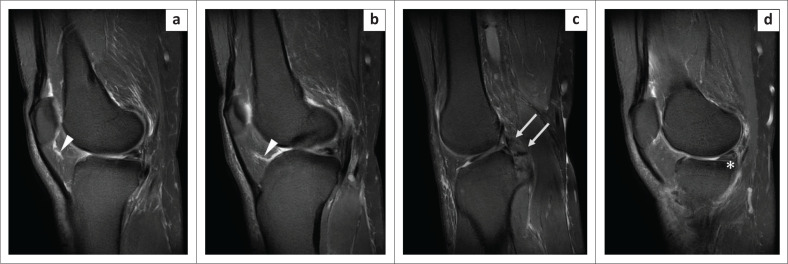
Magnetic resonance imaging of an infrapatellar plica tear in a 46-year-old male with a history of falling from a bike and knee pain: Serial oblique sagittal PDFS images (a) and (b) demonstrate a hyperintense fluid collection along the entire course of infrapatellar plica (arrow heads), with ragged margins. Oblique sagittal PDFS image (c) revealed that the patient also had an avulsion injury of the posterior cruciate ligament (arrows). Sagittal PDFS (d) indicated a complex tear of the posterior horn of the medial meniscus (asterisk).

**FIGURE 9:**
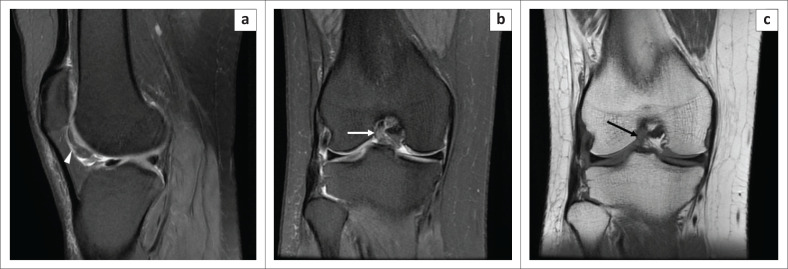
Magnetic resonance imaging of an infrapatellar plica tear in a 33-year-old patient with a twisting injury to the knee and clinically-suspected anterior cruciate ligament (ACL) injury: oblique sagittal PDFS image (a) revealed a hyperintense fluid collection along the entire course of the infrapatellar plica (arrow head), with irregular margins. Coronal PDFS image shows hyperintense signal along the ACL with intact fibres suggestive of mild sprain in the postero-lateral bundle of the ACL (arrow). Corresponding signal alteration on T1WI is shown in (c-arrow).

Plica injuries can be either a sprain or tear:

- **Sprain**: Increase in signal along the IPP on fluid-sensitive sequences, not corresponding to (less than) fluid signal intensity (Figures 4 and 5).
- **Tear**: Increased signal along the course of IPP having fluid signal intensity on fluid-sensitive sequences (Figures 6–9).

In spite of plica injury being a known entity in both the orthopaedic and radiology literature, it continues to remain under-reported and often neglected as a cause of anterior knee pain. An abnormal appearing plica in Hoffa’s fat pad by MRI may alert the orthopaedic surgeon to look for abnormality in the segment of IPP anterior to ACL. It is important to mention plica injuries in the MRI report because with timely diagnosis, treatment in the form of arthroscopic resection can be offered to the patient, especially if conservative treatment fails.^2,10,11^

Although often associated with other ligamentous and meniscal injuries that contribute to internal knee derangements (Figures 7–9), it is noteworthy that plica tears may be the only abnormality detected by MRI to explain the patient’s symptoms (Figures 4 and 6). Plica tear can often co-exist with ACL injuries, as illustrated in the case shown in Figure 9.

In the absence of any internal derangements and presence of MR evidence of plica abnormality, plica injury should be considered as the potential cause of anterior knee pain.^10,11^ Even if associated with other injuries, an injured IPP merits attention and resection on arthroscopy to prevent persisting pain.

## Plica tear mimickers

**FIGURE 10:**
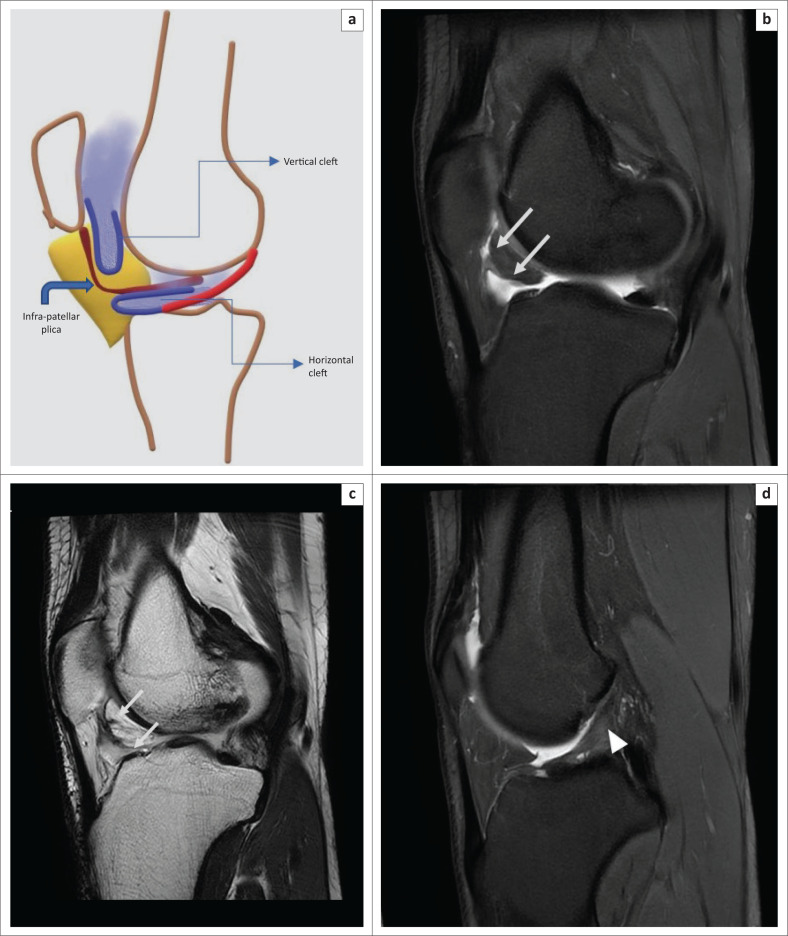
Magnetic resonance imaging of normal synovial clefts in Hoffa’s fat pad: schematic diagram (a) of the vertical (supra-Hoffotic) cleft and horizontal (infra-Hoffotic) cleft in relation to the infrapatellar plica. Magnetic resonance imaging, images (b–d) of normal supra-Hoffotic and infra-Hoffotic recesses in a 29-year-old boy with a history of a fall and clinically-suspected anterior cruciate ligament (ACL) injury. Oblique sagittal PDFS image (b) and oblique sagittal T2WI image (c) indicate hyperintense fluid-filled synovial clefts, contiguous with the joint fluid. Note the mild sprain in middle third of the ACL in (d), sagital PDFS (arrowhead).

**FIGURE 11:**
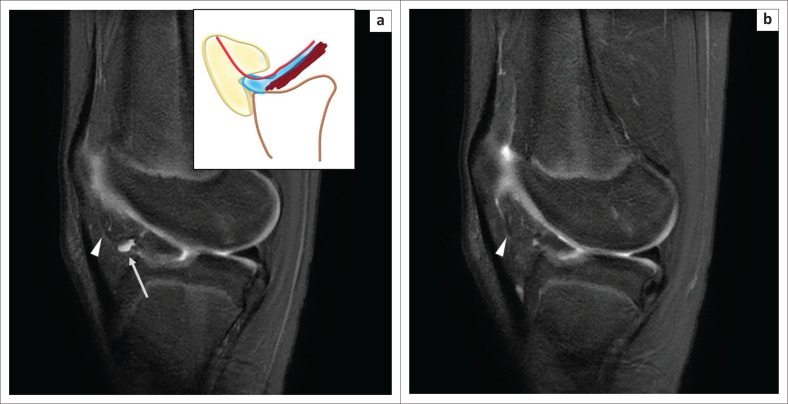
Magnetic resonance imaging of a normal horizontal cleft in Hoffa’s fat pad in a 14-year-old girl with a history of a fall: oblique sagittal PDFS image (a) shows the fluid-filled cleft (arrow) inferior to the infrapatellar plica (arrow head) in the posterior part of Hoffa’s fat pad, communicating with the joint cavity. The schematic representation of the horizontal cleft is shown in blue in the inset in (a). Oblique sagittal PDFS image (b) shows the separate intact infrapatellar plica (arrow head). Note the anterior cruciate ligament avulsion fracture which was the cause of pain in this case.

The abnormal signal in Hoffa’s fat pad along the IPP may also be found in the following conditions:

- **Synovial clefts in Hoffa’s fat pad:** Two synovium-lined clefts that communicate with the knee cavity are present along the posterior margin of the IPP, a vertical cleft located superiorly and a larger horizontal cleft located inferiorly. These can be identified in up to 70% and 90% of knee MRI examinations, respectively.^5,12^ The horizontal cleft is continuous with the joint cavity and its roof is formed by the IPP (Figures 10 and 11). It is not pathological, unless associated with ganglion cysts, loose bodies or other synovial pathologies like nodular synovitis or pigmented villonodular synovitis (PVNS). Horizontal synovial clefts can be differentiated from an IPP tear, which has a more cephalad extension up to the inferior pole of patella.^3,13^ Moreover, in our experience, the hyper-intensity in the case of sprain or plica tear typically has ragged/irregular margins as opposed to synovial clefts which are smoothly marginated and follow a continuous curvilinear pattern along the entire course of IPP (compare Figures 10 and 11 with Figures 6–9). Sometimes one may be able to appreciate an intact plica separately in the case of synovial clefts (Figure 11).
- **Post traumatic clefting/fragmentation of Hoffa’s fat pad:** Fluid-filled clefts may be noted within Hoffa’s fat pad related to knee trauma.^12^ However, the presence of a curvilinear shaped hyper-intensity, limited to the course of the IPP, favours a plica injury, often associated with Hoffa’s fat oedema and injury.

## Other causes of symptomatic plica

- **Plica syndromes:** Plica syndrome refers to painful impairment of knee function in which the only finding that helps explain the symptoms is the presence of a thickened and fibrotic plica, which becomes unyielding and snaps against the femoral condyle with repeated movement leading to erosive changes, degeneration and softening of the articular cartilage, resulting in synovitis or chondromalacia.^1,2^ The inciting event causing thickening of the plica may be acute trauma, repetitive stress injury, meniscal tears, osteochondritis dissecans, or loose bodies. The medial patellar plica is most likely to cause plica syndrome, followed by the superior plica. Infrapatellar plica may rarely be associated with trochlear groove chondromalacia.^4^
- **Imperforate superior plica** may lead to loculation of joint effusion in the suprapatellar recess and the patient may present with soft tissue swelling in this region.^3^

## Conclusion

Infrapatellar plica injuries are often overlooked as a cause of anterior knee pain and appear to have greater prevalence, much higher than previously expected. It is essential to include such injuries in knee MRI reports so that the surgeon can be alerted to arthroscopically remove the torn or abnormally-positioned plica which may otherwise continue to cause discomfort to the patient.

The limitation of this review is the lack of arthroscopic correlation. However, our observations open avenues for further research in a larger cohort of patients with arthroscopic correlation to confirm the same.

### Teaching points

- Infrapatellar plica injury is an under-reported cause of anterior knee pain and merits attention of the reporting radiologist as it is a treatable condition.
- Normal synovial clefts within Hoffa’s fat pad can mimic an IPP tear and can be differentiated based on location and margins. In plica tear, fluid signal within the Hoffa’s fat pad extends throughout the curvilinear course of IPP, has ragged margins, often with oedematous changes in the surrounding fat, which is not seen along synovial clefts.
- Plica injury can clinically mimic an ACL injury and the two often co-exist. Hence, in all cases of an ACL injury, an IPP tear should be sought for.
